# Four Complete *Paenibacillus larvae* Genome Sequences

**DOI:** 10.1128/genomeA.00407-17

**Published:** 2017-06-01

**Authors:** Douglas W. Dingman

**Affiliations:** Department of Entomology, The Connecticut Agricultural Experiment Station, New Haven, Connecticut, USA

## Abstract

Four complete genome sequences of genetically distinct *Paenibacillus larvae* strains have been determined. Pacific BioSciences single-molecule real-time (SMRT) sequencing technology was used as the sole method of sequence determination and assembly. The chromosomes exhibited a G+C content of 44.1 to 44.2% and a molecular size range of 4.29 to 4.67 Mbp.

## GENOME ANNOUNCEMENT

The Gram-positive bacterium *Paenibacillus larvae* causes American foulbrood and powdery scale disease in honey bee larvae (*Apis mellifera*). To increase the complete genome database for this bacterium, genomic DNA obtained from *P. larvae* subsp. *larvae* strain ATCC 9545^T^ and *P. larvae* subsp. *pulvifaciens* strains (ATCC 13537^T^, CCM 38, and SAG 10367) was sequenced and assembled. Strains ATCC 9545^T^ and ATCC 13537^T^ represent the type strains for their respective subspecies (1). DNA was extracted using the Qiagen Genomic-tip 500/G kit, according to the manufacturer’s protocol (Qiagen, Inc., Valencia, CA) and was sequenced and assembled at the Yale Center for Genomic Analysis (Yale University, New Haven, CT). Complete genomes were produced using only Pacific BioSciences (Menlo Park, CA) single-molecule real-time (SMRT) sequence reactions conducted on a PacBio RSII system. The SMRT Analysis software was used for secondary analysis, and the resulting assemblies were manually analyzed using Artemis (2) to correct and trim overlapping sequence on the ends. Annotation of the submitted genome assemblies was performed by NCBI staff using the NCBI Prokaryotic Genome Annotation Pipeline (https://www.ncbi.nlm.nih.gov/genome/annotation_prok/). Raw PacBio RSII read data have been submitted to the NCBI Sequence Read Archive (SRA).

Physical mapping of the chromosomes, based on I-CeuI endonuclease site locations, supported the accuracy of sequence assembly. I-CeuI recognizes a 29-bp DNA sequence within the 23S rRNA gene (3) and, by sequence orientation, can be used to determine the rRNA operon transcriptional direction. The presence of 7 I-CeuI sites in several *P. larvae* strains (6 *P. larvae* isolates in Connecticut and strain NRRL B-3650) has been reported (4). All 4 assembled chromosomes exhibited 8 I-CeuI sites (i.e., 8 copies of rRNA gene operons) with a transcriptional orientation ratio of 5:3. The origin of replication is presumed to occur between the rRNA operon transcriptional orientations. A G+C range of 44.1 to 44.2% and a molecular size range of 4.29 to 4.67 Mbp for these 4 chromosomes compared to genome reports for *P. larvae* in the NCBI GenBank database.

Sequence regions suggestive of bacteriophage DNA were identified in strains ATCC 13537^T^, CCM 38, and SAG 10367. These bacteriophage sequences in strains ATCC 13537^T^ and CCM 38 are of unknown origin. However, DNA sequence homologous to bacteriophage Sitara and Tripp (5, 6) was observed in strain SAG 10367. In addition, extrachromosomal DNA was found in these 3 strains and determined to assemble as circular molecules. The molecular sizes of the elements were quite large, and a homologous sequence was not found in the chromosomes. Although identified as plasmids in the NCBI GenBank database, they have not been confirmed. The extrachromosomal elements in strains ATCC 13537^T^ and CCM 38 exhibited the bacterial insertion sequence element IS*256*, implying the possibility of conjugative transfer capability. The chromosomes and extrachromosomal DNA within strains ATCC 13537^T^ and CCM 38 exhibited significant similarity, suggesting that these 2 strains are very closely related.

### Accession number(s).

The complete genome sequences of the *Paenibacillus larvae* strains are available at NCBI GenBank and have the following accession numbers: ATCC 9545^T^, CP019687; ATCC 13537^T^, CP019794 (pPLP1, CP019795; pPLP2, CP019796); CCM 38, CP020327 (pPLP1, CP020328; pPLP2.1, CP020329); and SAG 10367, CP020557 (pPLP3, CP020558).
